# Editorial: Functions, working mechanisms, and regulation of rotary ATPases and Ductin proteins

**DOI:** 10.3389/fmolb.2024.1399421

**Published:** 2024-03-28

**Authors:** Tibor Páli, Boris Feniouk, Stephan Wilkens

**Affiliations:** ^1^ Institute of Biophysics, HUN-REN Biological Research Centre, Szeged, Hungary; ^2^ A.N. Belozersky Institute, Lomonosov Moscow State University, Moscow, Russia; ^3^ Department of Biochemistry and Molecular Biology, State University of New York Upstate Medical University, Syracuse, NY, United States

**Keywords:** rotary enzyme, membrane, ATP hydrolysis, ATP synthesis, ATPase, *c*-ring, reversible disassembly, Ductin protein

## 1 Remaining challenges on rotary enzymes

The rotary mechanism of the ion-transporting F-, V- and A-type ATPases is of great interest to the molecular bio-sciences. Connecting the regulation of their rotation-coupled catalysis-transport cycle with the various associated biological functions requires a detailed understanding of the enzymes’ rotary mechanism. Despite recent progress with developing new and alternative models [see, e.g., Frasch et al. (2022); Kishikawa et al. (2022); Nakano et al. (2023); Nath (2023)], a full understanding of the biological functions of these enzymes is limited by the fact that measuring the proton-transfer and rotation rates of rotary ATPases in the cellular context still represents a significant challenge. For instance, the native rotation rate of a chemically intact enzyme could be measured so far only using indirect methods (Ferencz et al., 2013; Ferencz et al., 2017; Petrovszki et al., 2021). Though recent cryo electron microscopy (cryo-EM) studies have provided near-atomic snapshots for some of the ion-transporting subcomplexes [e.g., Kishikawa et al. (2022); Pinke et al. (2020)], the challenge also remains that the “substrate”—protons—cannot be visualised directly. There is accumulating evidence that, as part of the Ductin family (Holzenburg et al., 1993; Lautemann and Bohrmann, 2016), some rotor or “*c*-ring” proteins are key players in certain membrane fusion and rearrangement processes even in the absence of the catalytic activity of the holoenzyme [see, e.g., Higashida et al. (2017); Rama et al. (2019); Amodeo et al. (2021); Lévêque et al. (2023)]. However, it is challenging to separate the physiological role of the isolated *c*-ring proteins from their role in the intact enzyme in those processes. This Research Topic gathered valuable articles presenting new data and views on the molecular mechanisms and physiological roles of these membrane-transporter rotary enzymes. The key findings of these articles are summarised in the next two sections.

## 2 On the catalytic and transport mechanisms of the rotary ATPases

Based on extensive time-resolved cryo-EM snapshot analyses, Yokoyama’s review provides a comprehensive overview of the structure and function of the rotary V/A-ATPase from the thermophilic bacterium *Thermus thermophilus*, one of the best characterised rotary ATPases. The authors of the study conclude that the rotary mechanism of the related F_1_-ATPase is more complex than that of the V/A-ATPase (regarding the events of ATP binding and hydrolysis coupled rotation), but also that the underlying principle is conserved. Suiter and Volkán-Kacsó analysed (at microsecond time-resolution) single-molecule rotational trajectories of F_1_-ATPase of a bacterial species, *Paracoccus denitrificans*, imaged by a nano-crystal probe attached to the rotor shaft of the motor (these data were generated by Noji and coworkers). They found a common mechanism for removing a nucleotide release bottleneck in the rotary mechanism in the *P. denitrificans* and *Thermophilic bacillus* F_1_-ATPase. The paper also discusses how the F-ATPase was perfected by evolution for efficient and robust energy conversion. In another single-molecule study Yanagisawa et al. present rotation-experiments carried out with high-resolution of time and rotational angle for the V_1_ subcomplex of the yeast, *Saccharomyces cerevisiae* V-ATPase. The results provide great detail on the molecular basis for the differences in rotor positions associated with substrate binding and product release between V- and F-type ATPases. A radically new theory that departs from the concept of the chemo-mechanical coupling (transduction of chemical free energy of ATP to mechanical work) for an ATP-driven protein complex is presented by Yasuda et al. According to the authors of the study, the entropy originating from the displacement of water molecules in the system plays a key role in driving rotation. The paper concludes that ATP hydrolysis (or synthesis) is tightly coupled to the rotation of the central shaft in the normal (or inverse) direction through a water-entropy effect.

## 3 On the biological functions and regulation of rotary ATPases and Ductin proteins

Tuli and Kane’s review provides strong arguments for why the cytosolic N-terminal domain of the *a*-subunit of V-ATPases functions as a regulatory hub for enzyme targeting via multiple signals. One such regulatory mechanism, binding to phosphoinositides, targets mammalian *a*-subunit isoforms to specific membranes, and regulates the enzymes’ ATP hydrolysis and proton pumping activities. The study of Mendoza-Hoffmann et al. presents a sizeable amount of (bioinformatic, biochemical, molecular biology, functional and structural) data about the evolution and regulatory role of the *ζ*-subunit of the F-ATPase of *P. denitrificans* and *α*-proteobacteria. It is convincingly argued that the *ζ*-subunit evolved by preserving its inhibitory function in free-living *α*-proteobacteria, however, this function was lost in some symbiotic *α*-proteobacteria where it became non-essential given the possible exchange of nutrients and ATP with the host. The report of Wang et al. relates to the role of V-ATPase in synaptic vesicle neurotransmitter loading and in vesicle fusion, and it is considered as an ideal candidate to regulate the fusogenic status of secretory vesicles according to their loading state. Their experimental results argue that, via V_*o*_-V_1_ dissociation, V-ATPase modulates exocytosis in neuroendocrine cells through the activation of the synthesis of phosphatidic acid. And finally, Sebők-Nagy et al. hypothesise that binding of divalent cations to the *c*-ring, or more generally Ductin protein assemblies, acts as a new regulatory mechanism of certain membrane trafficking processes. The authors of the study propose that such non-covalent binding of certain divalent cations could structurally modulate the various functions of Ductin assemblies by affecting their stability.

## 4 Perspectives

Structural biology of membrane proteins is rapidly catching up thanks to improved experimental approaches (for example, cryo-EM) (e.g., Pinke et al., 2020; Gerle et al., 2022; Yamamori and Tomii, 2022) and structure predictions enhanced with artificial intelligence (Versini et al., 2023; Wuyun et al., 2024). Structure models with atomic detail are already available, also for the F_*o*_, V_*o*_ and A_*o*_ domains. The improved structure models combined with kinetic single-molecule spectroscopic and other novel biophysical studies (e.g., Otomo et al., 2022; Kobayashi et al., 2023; Pérez et al., 2023) and molecular simulations (e.g., Blanc and Hummer, 2024) will lead to more detailed theoretical description of the catalysis-transport mechanism of F-, V- and A-type rotary enzymes. Regarding biological function, research is strong on the assembly and the activity of the rotary enzymes (and some of their subunits, e.g., *c*-ring proteins) in general, but also in certain membrane fusion and pore formation processes (Novitskaia et al., 2019; Banerjee and Kane, 2020; Mnatsakanyan and Jonas, 2020; Abuammar et al., 2021; Lapashina et al., 2022; Nesci, 2022; Wilkens et al., 2023; Yamamoto et al., 2023). Therefore, new insights will likely emerge on the biological regulation of the reversible assembly and activity of rotary enzymes in the near future.
